# Predictors of the intention to use traditional Chinese medicine (TCM) using extended theory of planned behavior: a cross-sectional study among TCM users in Hong Kong

**DOI:** 10.1186/s12906-022-03598-x

**Published:** 2022-04-22

**Authors:** Tommy K. C. Ng, Man Fung Lo, Ben Y. F. Fong, Hilary H. L. Yee

**Affiliations:** 1 School of Professional Education and Executive Development, College of Professional and Continuing Education, The Hong Kong Polytechnic University, Kowloon, Hong Kong; 2 Faculty of Social Science, The Chinese University of Hong Kong, New Territories, Hong Kong

**Keywords:** Traditional Chinese medicine, Theory of planned behavior, Attitude

## Abstract

**Background:**

Traditional Chinese medicine (TCM) and Western medicine are available and have a long history in Hong Kong. Little is known on the intention to use TCM in Hong Kong. In this study, the intention to use TCM was examined by the extended theory of planned behavior.

**Methods:**

An online cross-sectional survey was conducted from 15 and 29 November 2021. Respondents’ attitude towards using TCM, intention to use TCM, knowledge, perceived behavioral control, perceived service quality, satisfaction, subjective norms and demographic characteristics were collected from the survey. To examine the conceptual framework in this study, partial least square structural equation model (PLS-SEM) was adopted.

**Results:**

In total, 446 responses (completion rate = 74.3%) were analysed. Attitude towards TCM was the strongest predictor to the intention to use TCM followed by satisfaction and subjective norms. Attitude had a partial mediating effect on the relationship between subjective norms, perceived behavioral control and intention to use TCM. Attitude had a full mediating effect on the relationship between knowledge and intention to use TCM. Satisfaction had a full mediating effect on the relationship between perceived service quality and intention to use TCM.

**Conclusions:**

This is the first study to investigate the predictors of the intention to use TCM in Hong Kong by using TPB. Individuals’ attitude towards TCM has showed stronger effect to the intention to use TCM than other predictors, such as satisfaction, perceived behavioral control and subjective norms. To enhance the intention to use TCM in Hong Kong, key stakeholders, including TCM professional organisations, health professionals and policymakers, should develop a positive attitude towards TCM among Hong Kong population.

**Supplementary Information:**

The online version contains supplementary material available at 10.1186/s12906-022-03598-x.

## Introduction

Traditional Chinese medicine (TCM) has a long history in China and it has a great potential for health improvement, prevention and treatment. Western medicine is the mainstream medical practice in Hong Kong. Hong Kong was a British colony from 1841 to 1997, before the handover of sovereignty back to China. At the beginning of colonial years, TCM remained popular because of distrusting Western medicine by people [1]. However, the advancement of Western medicine, increased price of TCM and time consuming for the preparation of herbs had caused the decline in popularity of TCM after the Second World War. With proper regulation and registration of TCM, the development of TCM is advancing in the last decades. Both TCM and Western medicine are available and popular medical practice in Hong Kong.

The importance of TCM in primary care has been recognised, with 50–60% of the Hong Kong population consulted TCM practitioner at least once in their lives [2]. There are some evidences showing that TCM can be an effective alternative to Western medicine. TCM, consists of herbal medicine, acupuncture, moxibustion and massage, which have the positive effects on pain management, palliative care and management of rheumatoid arthritis [3–6]. Besides, the use of TCM can alleviate the side effects and optimise the clinical effectiveness of radiotherapy and chemotherapy [7]. For example, radiotherapy and chemotherapy for cancer treatments are commonly causing different side effects like fatigue, vomiting, nausea, hair loss, skin ulcers, etc. [7]. TCM can help relieving the side effects caused by radiation and optimize the clinical effectiveness of radiotherapy and chemotherapy, specifically to guide the tonifying qi and producing blood, to reinforce the spleen and stomach and to eliminate heat produced by blood and toxic substance inside the body [7]. TCM and integrated medicine may improve the quality of care and life of the patients. In Hong Kong, elderly and female are more likely to utilise Chinese medicine primary care and patients using TCM have the aim to manage their chronic diseases [2]. Therefore, the preference of TCM is mainly the management of chronic conditions.

Previous studies have investigated the effectiveness of using TCM on disease management, disease prevention and health maintenance [3–6, 8]. However, to the best knowledge of the researchers, no comprehensive studies have investigated the predictors of the intention to use TCM in Hong Kong. This study aimed to use the extended theory of planned behavior to identify the predictors of the intention to use TCM. The findings of this study could provide new insights for policymakers, practitioners and researchers in promoting TCM to the public. Therefore, the research question for this study was: What factors are predicting the intention to use TCM in Hong Kong?

### Literature review

#### Theory of planned behavior

The Theory of Planned Behavior (TPB) is a well-known model in explaining health-related behavior. TPB has been used in many research, including the evaluation of smoking behavior interventions [9], adoption of social distancing [10], COVID-19 vaccination intention [11] and mobile health service adoption [12], and so TPB is a well-established and validated model. In TPB, behavioral intention, which is affected by subjective norms (perceived expectations from important people on performing the behavior), attitude (personal feelings and beliefs about the behavior) and perceived behavioral control (perceived ability of performing the behavior), is the predictor of actual behavior [13–15].

Subjective norms relates to the likelihood that the important people will agree or disagree of certain behavior and personal motivation to meet their expectations [16]. In some studies, subjective norms has a positive effect to the attitude towards using TCM. Source of TCM information are mostly from family, friends and Chinese medicine practitioner [17]. More than 40% of the medical students in Hong Kong would like to use TCM because of the recommendation from family and friends [18]. Subjective norms significantly predicted attitude towards the use of TCM in China [19]. Additionally, recommendations and referrals from those who are trustworthy could motivate the adults to use acupuncture in Hong Kong [20]. Therefore, the following hypotheses are developed:

- *H1: Subjective norms is positively related to the attitude towards using TCM.*
- *H2: Subjective norms is positively related to the intention to use TCM.*

Perceived behavioral control is about the perceived ability to perform the behavior. The easier to perform the behavior leads to the increase in attitude and intention to perform the behavior. Attitude towards the use of TCM was found to be affected by perceived behavioral control [21]. Some studies have found that perceived behavioral control is one of the predictors of acquiring knowledge intention towards and the use of complementary and alternative medicine [22, 23]. Likewise, perceived behavioral control was found to significantly predict the intention to use TCM in China [24]. Therefore, this study proposes hypothesis 3 and 4 as below:

- *H3: Perceived behavioral control is positively related to the attitude towards using TCM.*
- *H4: Perceived behavioral control is positively related to the intention to use TCM.*

Positive attitude towards behaviors is correlated to the intention to the behaviors. The relationship between the attitude towards behaviors and intention to the behavior was found in many studies using TPB, including wearing PM2.5 mask [25], consumer purchasing behavior for organic food [26], and self-management of stroke [27]. Individual’s attitude towards herbal medicines consumption was correlated with intention of herbal medicine use among pregnant women [28]. Besides, attitude was found to significantly predict the intention to use TCM for health maintenance [24]. Positive attitude about traditional, complementary and alternative medicine had a positive relationship with the willingness to pay for the medicine [29]. The following hypotheses are thus proposed:

- *H5: Attitude towards using TCM is positively related to the intention to use TCM.*
- *H6. Attitude towards using TCM mediates the relationship between subjective norms and the intention to use TCM.*
- *H7. Attitude towards using TCM mediates the relationship between perceived behavioral control and the intention to use TCM.*

#### Knowledge, attitude and behavioral intention

Individual’s knowledge about the behavior can be one of the predicting factors of attitude towards behavior and behavioral intention. Knowledge is important to provide meaningful and useful information for individuals. Knowledge can be viewed as a distal antecedent of behavior conveyed by attitude [30]. Knowledge is important to the attitudes towards health behaviour under the knowledge-attitude-behaviour model [31]. Patients’ attitude towards using acupuncture had a positive impact on the attitude towards acupuncture, affected by their knowledge [32]. Individual with sufficient knowledge can understand the safety and risk of performing behavior so the level of knowledge may affect the behavioral intention. Abamecha et al. had found that knowledge about cervical cancer was positively associated with intention to use cervical cancer screening [33]. Therefore, the following hypotheses are developed:

- *H8: The knowledge of TCM is positively related to the attitude towards using TCM.*
- *H9: The knowledge of TCM is positively related to the intention to use TCM.*
- *H10. Attitude towards using TCM mediates the relationship between the knowledge of TCM and the intention to use TCM.*

#### Perceived service quality, satisfaction and behavioral intention

In some studies, customer satisfaction theory was used to predict the relationship of perceived service quality, satisfaction and behavioral intention [34, 35]. Perceived service quality refers to the comparison of prior expectations with perceived performance of a specific service [36, 37]. Satisfaction is interpreted as patient treatment satisfaction, clinical experience and service recommendation [38, 39]. The relationship between perceived service quality, patient satisfaction and behavioral intention was found in many studies [34–37, 40–42]. Therefore, the following hypotheses are developed:

- *H11: The perceived service quality is positively related to the intention to use TCM.*
- *H12: The perceived service quality is positively related to the satisfaction of using TCM.*
- *H13: The satisfaction of using TCM is positively related to the intention to use TCM.*
- *H14: The satisfaction of using TCM mediates the relationship between the perceived service quality and the intention to use TCM.*

**Fig. 1:**
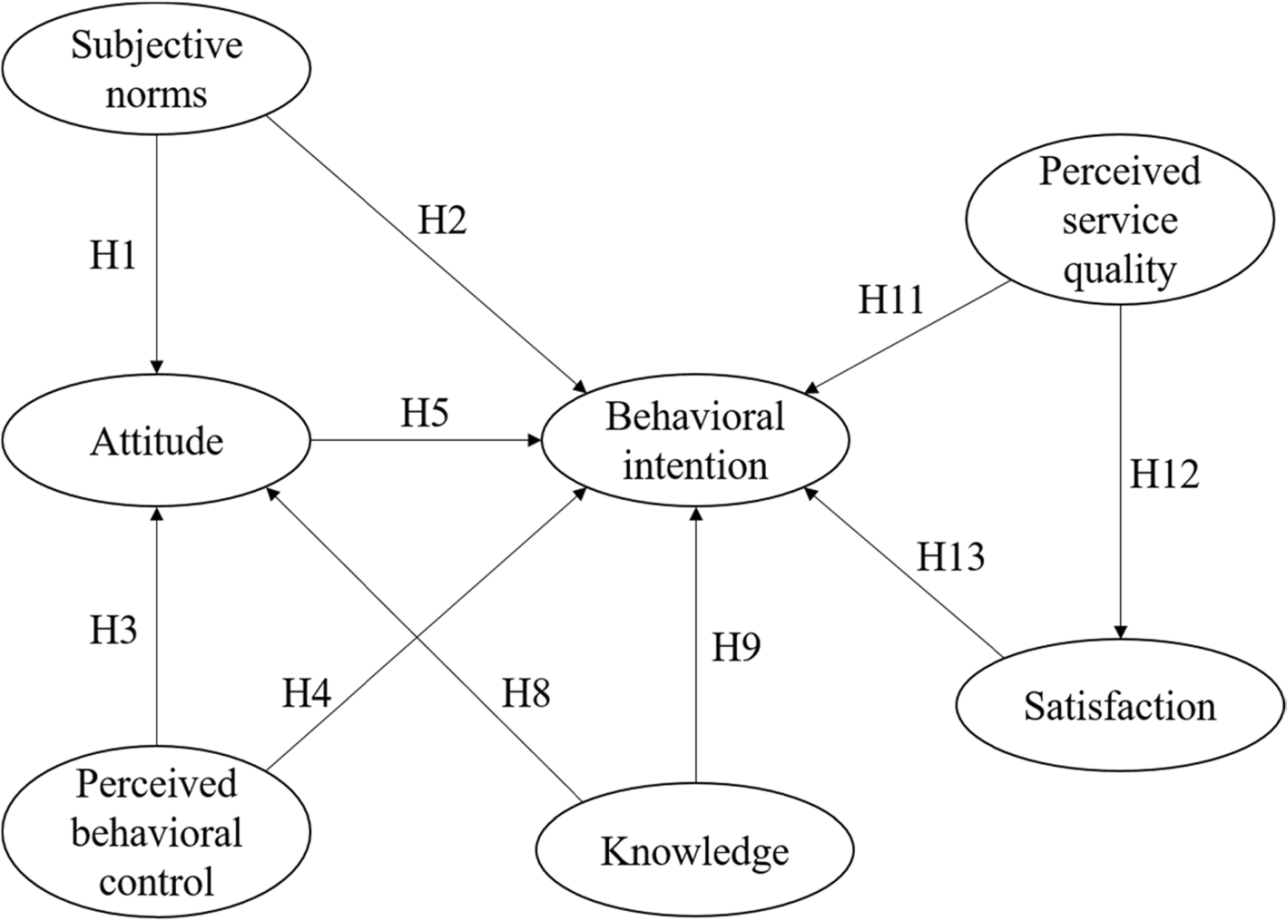
Conceptual model

A conceptual model which depicts the relationships between the constructs of TPB, knowledge, satisfaction and perceived service quality of TCM has been developed (Fig. 1).

### Methodology

#### Participants and recruitment procedure

This online cross-sectional survey by a questionnaire was conducted between 15 and 29 November 2021 to address the research question. This study was approved by the Research Committee of College of Professional and Continuing Education, The Hong Kong Polytechnic University. The methodology and results of this study were reported in accordance with the Checklist for Reporting Results of Internet E-Surveys (CHERRIES) [43].

The online questionnaire was initially and purposely sent to participants with varying age and educational level. The link of the survey was posted on social media platforms hosted by the academic institution. Participants were encouraged to forward the questionnaire link to relatives and friends whom they considered suitable. Participants who were literate in Chinese and able to understand the questionnaire; Hong Kong residents aged 18 or above; could access to the internet via smart-phone or computer; consented to participating before they could start the survey; and had used TCM before were included in this study. Interested subjects would read a statement describing the background of the survey, anonymity assurance and participation agreement at the beginning of the survey. Checking the consent box represents that they gave their agreement for study participation. Participation was optional and anonymous. To avoid multiple submissions, the prefix and first three digits of Hong Kong Identity Card (HKID) were collected in the questionnaire. Participants could leave their email address if they were interested to be contacted for survey clarification or join future follow up studies. Identifiers, such as HKID, were removed before data analysis. All decisions were made at subjects’ own discretion. No financial incentives were given after the completion of the questionnaire. The calculation of target sample size should be 10 times the maximum number of measuring items pointing at the construct in the PLS path model [44]. In the present study, there are 33 items measuring seven constructs so the target sample size is 330 (10 × 33).

#### Measures of questionnaire

The questionnaire consisted of four parts to collect the data regarding the attitude, subjective norms, perceived behavioral control, intention, knowledge, perceived service quality and satisfaction of TCM of respondents. All items in the TPB framework and satisfaction of the use of TCM were measured using the 7-point Likert scale (1 = strongly disagree / Never to 7 = strongly agree / Always). For the perceived service quality, a validated short questionnaire to assess perceived service quality was used [45]. Five items regarding perceived service quality was asked using the 7-point Likert scale (1 = strongly disagree to 7 = strongly agree). The knowledge construct was measured by four questions regarding their self-report knowledge of basic theory, medicine, diagnosis and diet using the 7-point Likert scale (1 = strongly disagree to 7 = strongly agree). The questionnaire items of the seven constructs in this study is shown in Supplementary file 1. Several demographic questions (i.e., age, gender, level of education, economic status, religion, and self-report health status) were also asked in the survey. Respondents were allowed to select more than one religion in the questionnaire.

#### Data analysis

To examine the conceptual framework in this study, the partial least squares structural equation model (PLS-SEM) was adopted. The reflective measurement model and structural model were examined with the SmartPLS 3.0 statistical software. The reliability and validity of the measurement instrument were verified. To evaluate the reliability of this study, value of Cronbach’s alpha and composite reliability greater than 0.7 is considered to be reliable [44]. The convergent validity is considered good if the outer loadings of the measurement items are greater than 0.6 and the Average Variance Extracted (AVE) of each construct greater than 0.5 [44, 46]. To evaluate discriminant validity, both Fornell Larcker criterion and heterotrait-monotrait (HTMT) ratio were assessed.

## Results

### Participant characteristics

There were 446 valid responses (*n* = 446/600, completeness rate: 74.3%). Table 1 shows the demographic characteristics of the participants. 38.6% of the respondents were aged 18 to 25 and 60.3% of the respondents were female. Most of the participants (85.2%) had attained a post-secondary educational level or above. Nearly half and one third of the participants had rated their health status as fair and good respectively.

**Table 1:** Demographic characteristics

Variables	Number of respondents	Percentage
**Age**		
18–25	172	38.6
26–35	51	11.4
36–45	55	12.3
46–55	47	10.5
56–65	72	16.1
66–75	45	10.1
76 or above	4	0.9
**Gender**		
Male	177	39.7
Female	269	60.3
**Educational level**		
Primary of below	3	0.7
Secondary	63	14.1
Post-secondary	169	37.9
Degree	113	25.3
Postgraduate or above	98	22.0
**Religion**		
Christianity	98	22.0
Catholic	22	4.9
Buddhism	49	11.0
Taoism	11	2.5
Confucianism	2	0.4
No religion	271	60.8
**Employment status**		
Student	168	37.7
Employed	170	38.1
Self-employed	21	4.7
Unemployed	8	1.8
Retired	79	17.7
**Monthly income (HK$)**		
≤ 4000	171	38.3
4001–16,000	55	12.3
16,000 – 28,000	81	18.2
> 28,000	139	31.2
**Health status**		
Poor	46	10.3
Fair	214	48.0
Good	145	32.5
Very good	33	7.4
Excellent	8	1.8

#### Measurement model

The reliability of the model is presented in Table 2. The variance inflation factor (VIF) level of each item was lower than 5, indicating no critical levels of collinearity [44]. All items had outer loadings of greater than 0.6, except one item of attitude construct and one item of perceived service quality. Hence, these two items were deleted. The composite reliability and Cronbach’s alpha were higher than the threshold value of 0.7, indicating a satisfactory internal consistency reliability. The AVE of all constructs were above 0.5, suggesting that the constructs had good convergent validity [44]. The results of the assessment of discriminant validity are presented in Table 3. The discriminant validity in this study was supported. With adequate reliability, convergent validity and sufficient discriminant validity, it was possible to move forward to assess the structure model.

**Table 2:** Loadings, Cronbach’s alpha, average variance extracted (AVE), and construct reliability (CR) of the measurement model

Constructs	Items	Mean	SD	VIF	Loadings	AVE	Composite reliability	Cronbach’s alpha
**Attitude (ATT)**						0.695	0.919	0.888
A_1_	I extremely trust traditional Chinese medicine (TCM)	5.50	1.070	2.856	0.883			
A_2_	I am very concerned about TCM	4.89	1.333	2.000	0.801			
A_3_	I’d very much like to accept TCM theory	5.56	1.149	2.997	0.887			
A_4_	I think TCM is effective	5.77	0.953	2.586	0.861			
A_5_	I think TCM is safe	5.62	0.959	1.618	0.725			
**Behavioral intention (BI)**						0.820	0.948	0.926
BI_1_	I would like to use TCM in the future	5.51	1.149	2.210	0.847			
BI_2_	I would recommend relatives, friends and colleagues to choose TCM	5.39	1.171	4.071	0.926			
BI_3_	I would say positive things about TCM to others	5.55	1.116	4.034	0.922			
BI_4_	I would encourage others to use TCM	5.36	1.208	4.336	0.925			
**Knowledge (K)**						0.798	0.940	0.916
K_1_	I know the basic theory of TCM	4.38	1.426	2.940	0.909			
K_2_	I know the medicine of TCM	3.98	1.410	2.973	0.891			
K_3_	I know the diagnosis of TCM	4.00	1.452	3.058	0.893			
K_4_	I know the diet of TCM	4.46	1.437	2.624	0.880			
**Perceived behavioral control (PBC)**						0.512	0.839	0.765
PBC_1_	I have time to receive TCM services	5.29	1.157	1.508	0.745			
PBC_2_	I am economically capable to receive TCM services	5.42	1.158	1.644	0.681			
PBC_3_	I have the ability to decide whether to choose TCM	5.93	0.913	1.508	0.664			
PBC_4_	I can share my knowledge and experience of TCM with others	5.22	1.306	1.525	0.766			
PBC_5_	I can overcome my difficulty in choosing TCM	5.18	1.233	1.407	0.716			
**Perceived service quality (PSQ)**						0.704	0.922	0.894
PSQ_1_	The TCM services provide their service at the time they promise to do so	5.22	1.090	1.792	0.746			
PSQ_2_	Personnel of the TCM services react promptly to my requests	5.12	1.087	2.342	0.845			
PSQ_3_	Personnel of the TCM services are polite	5.47	1.048	2.738	0.862			
PSQ_4_	Personnel of the TCM services give me personal attention	5.27	1.116	3.639	0.872			
PSQ_5_	Personnel of the TCM services communicate carefully with me	5.45	1.026	2.969	0.863			
**Satisfaction (SAT)**						0.847	0.957	0.940
S_1_	My chief complaints can be addressed after receiving TCM	5.23	1.130	3.907	0.916			
S_2_	I feel better after receiving TCM	5.43	1.093	4.493	0.934			
S_3_	TCM can fulfill my expectation of treatment	5.27	1.142	3.870	0.924			
S_4_	I am satisfied with the last curative effect	5.18	1.191	3.359	0.907			
**Subjective norms (SN)**						0.690	0.899	0.852
SN_1_	My family and friends support me to choose TCM	5.40	1.084	1.898	0.842			
SN_2_	My family and friends think I should choose TCM	4.83	1.094	2.136	0.825			
SN_3_	My family and friends will choose TCM	5.10	1.123	2.176	0.831			
SN_4_	My family and friends choose TCM, I would make the same choice	4.97	1.260	1.721	0.824

**Table 3:** Construct correlations, square roots of AVE (in italic) and HTMT values

	ATT	BI	K	PBC	PSQ	SAT	SN
ATT	*0.837*	0.851	0.451	0.702	0.535	0.725	0.713
BI	0.775	*0.906*	0.428	0.744	0.578	0.720	0.727
K	0.413	0.399	*0.894*	0.477	0.402	0.367	0.462
PBC	0.599	0.645	0.433	*0.718*	0.610	0.593	0.687
PSQ	0.475	0.526	0.366	0.514	*0.840*	0.535	0.558
SAT	0.663	0.673	0.344	0.514	0.492	*0.920*	0.567
SN	0.632	0.657	0.408	0.578	0.490	0.514	*0.830*

*ATT* Attitude, *BI* Behavioral intention, *K* Knowledge, *PBC* Perceived behavioral control, *PSQ* Perceived service quality, *SAT* Satisfaction, *SN* Subjective norms

#### Structural model

**Fig. 2:**
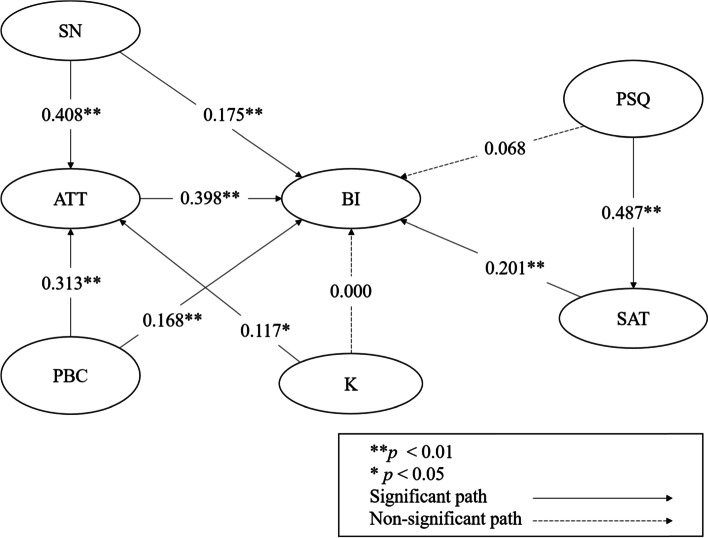
Results of structural model. ATT: Attitude; BI: Behavioral intention; K: Knowledge; PBC: Perceived behavioral control; PSQ: Perceived service quality; SAT: Satisfaction; SN: Subjective norms

The results of the direct effects of structural model are shown in Table 4. These results supported the hypotheses H1, H3 and H8, indicating that the subjective norms (*β* = 0.408; *p* <  0.01), perceived behavioral control (*β* = 0.313; *p* <  0.01) and knowledge of TCM (*β* = 0.117; *p* <  0.05) are positively related to the attitude towards using TCM but the result exhibited weak effect sizes on knowledge. The results showed that subjective norms (*β* = 0.175; *p* <  0.01), perceived behavioral control (*β* = 0.168; *p* <  0.01), attitude (*β* = 0.398; *p* <  0.01) and satisfaction (*β* = 0.201; *p* <  0.01) have significantly positive effects on the intention to use TCM. However, there was a non-significant relationship between knowledge and behavioral intention of using TCM (*β* = 0.000; *p* > 0.05) and perceived service quality and behavioral intention of using TCM (*β* = 0.068; *p* > 0.05). H12 stated that the perceived service quality was positively related to the satisfaction and the results provided statistical support for this hypothesis (*β* = 0.487; *p* <  0.01). The results of structural model are shown in Fig. 2. The mediation model results are shown in Table 5. All meditation hypotheses were supported. Attitude had a partial mediating effect on the relationship between subjective norms, perceived behavioral control and intention to use TCM. Attitude had a full mediating effect on the relationship between knowledge and intention to use TCM. Satisfaction had a full mediating effect on the relationship between perceived service quality and intention to use TCM.

**Table 4:** Results of the direct effects of structural model

Hypothesis	Path relations	β	*t* values	*p*-values	Decision
H1	SN ➔ ATT	0.408	9.226	< 0.01	Supported
H2	SN ➔ BI	0.175	4.128	< 0.01	Supported
H3	PBC ➔ ATT	0.313	6.360	< 0.01	Supported
H4	PBC ➔ BI	0.168	4.132	< 0.01	Supported
H5	ATT ➔ BI	0.398	7.718	< 0.01	Supported
H8	K ➔ ATT	0.117	2.249	< 0.05	Supported
H9	K ➔ BI	0.000	0.004	0.997	Not supported
H11	PSQ ➔ BI	0.068	1.705	0.088	Not supported
H12	PSQ ➔ SAT	0.487	9.379	< 0.01	Supported
H13	SAT ➔ BI	0.201	4.146	< 0.01	Supported

*ATT* Attitude, *BI* Behavioral intention, *K* Knowledge, *PBC* Perceived behavioral control, *PSQ* Perceived service quality, *SAT* Satisfaction, *SN* Subjective norms

**Table 5:** Results of the mediating effects of structural model

Hypothesis	Path relations	Indirect effect	*t* values	*p*-values	Result
H6	SN ➔ ATT ➔ BI	0.162	5.919	< 0.01	Partial mediating effect
H7	PBC ➔ ATT ➔ BI	0.125	5.085	< 0.01	Partial mediating effect
H10	K ➔ ATT ➔ BI	0.046	2.147	< 0.05	Full mediating effect
H14	PSQ ➔ SAT ➔ BI	0.098	4.065	< 0.01	Full mediating effect

*ATT* Attitude, *BI* Behavioral intention, *K* Knowledge, *PBC* Perceived behavioral control, *PSQ* Perceived service quality, *SAT* Satisfaction, *SN* Subjective norms

## Discussion

The study sought to assess and examine the relationships among attitude towards TCM, intention to use TCM, knowledge, perceived behavioral control, perceived service quality, satisfaction and subjective norms. The conceptual model was established based on the TPB by integrating customer satisfaction theory and knowledge-attitude-behaviour theory. The present study showed that the attitude towards TCM was significantly influenced by subjective norms, perceived behavioral control and knowledge. For example, advice and referrals by friends and health professionals would motivate individuals to use acupuncture [20]. Additionally, perceived behavioral control was positively related to attitude. Affordability and accessibility have been found to be the reason of using herbs among diabetes patients [47]. Moreover, this study showed that knowledge was positively related to the attitude towards TCM. Such positive correlation towards CAM has been found among health professionals and patients [48, 49]. Nevertheless, another study has not found a significant positive relationship between knowledge and attitude [50]. Further study to examine the knowledge and attitude relationship is needed.

The behavioral intention to use TCM was significantly affected by subjective norms, perceived behavioral control, attitude and satisfaction. This is consistent with the study by McIntyre et al. which demonstrated attitude, subjective norms and perceived behavioral control were significantly predicted the intention to use herbal medicines to manage anxiety symptoms, but strong association between perceived behavioral control and intention to use herbal medicines were found [51]. Nevertheless, a meta-analysis has concluded that attitude towards the behavior is the most important predictor of health behavior intention [52]. Furthermore, satisfaction was one of the predictors to the intention to use TCM. This finding is in line with that of another study which found that satisfaction with treatment was one of the reasons to use acupuncture [20]. The effectiveness of the treatment has influenced the compliance of participating in acupuncture trial [53]. Satisfying with the treatment may cause the users to continue the use of TCM. Our findings also provided empirical support for the mediating role of attitude towards TCM in TPB. Higher levels of subjective norms and perceived behavioral control increased the intention to use TCM directly but also increased individuals’ attitude, which in turn led to intention to use TCM. Therefore, some of subjective norms and perceived behavioral control’s effect on intention to use TCM was explained by attitude.

Contrary to the expectations, knowledge and perceived service quality did not have a significant direct influence on the intention to use TCM. Research has demonstrated that there is no association between patients’ familiarity with and willingness to pay for TCM services [54]. However, lack of knowledge with TCM is one of the concerns about undesirable effects among cancer patients [55]. Therefore, lack of knowledge about TCM may decrease patients’ intention to use TCM. Previous study has also found cancer patients’ willingness to use TCM for rehabilitation is positively correlated with their educational level [56], but another study has found no relationship between educational level and intention to use TCM [24]. Nevertheless, our findings provided empirical support for the mediating role of attitude in the knowledge and intention to use TCM relationship. The results indicated that knowledge affected attitude, and attitude in turn led to intention to use TCM. On the other hand, the result of this study could not find statistically significant direct effect between perceived service quality and intention to use TCM. Unlike a previous study that had demonstrated perceived service quality has a positive effect on repurchasing and sharing of TCM [57]. The mediating role of satisfaction in the relationship between perceived service quality and intention to use TCM was found in the present study. Thus, perceived service quality leads to satisfaction, and satisfaction in turn leads to intention to use TCM.

### Practical implication

Predicting the intention to use TCM may facilitate the promotion of TCM in Hong Kong. This study has concluded that attitude towards TCM has the strongest effect on the intention to use TCM, while satisfaction to the TCM is the second predictor to the intention of using TCM. The findings may assist key stakeholders, including TCM profession and related organisations, health professionals and policymakers, to develop policies and community strategies for promoting positive attitude towards TCM and enhancing service quality. Besides, subjective norms and perceived behavioral control could influence individuals’ attitude towards TCM, and lead to positive intention to TCM. To promote the use of TCM in Hong Kong, the development of evidence-based TCM is important. Evidence-based TCM allows both practitioners and patients to understand the effectiveness of TCM and more widely accept, recognise and apply TCM [58]. The integration of TCM and Western medicine may facilitate the promotion of TCM in Hong Kong. Public-private partnership on the use of TCM can foster the integration of TCM and Western medicine. Additionally, building the reputation of the TCM practitioners may enhance the intention to use TCM. TCM practitioners need to take this into account that the quality of treatment is vital for TCM users to have revisit intention to use TCM.

### Research implication

The present study offers theoretical contributions to researchers because it is the first study using the extended theory of planned behavior to examine predictors of intention to use TCM. The findings of this study could enhance researchers’ understanding on how TCM users’ intention to use TCM was influenced by different factors. The results may provide insights for researchers to conduct future research on exploring the in-depth views on the intention to use TCM among TCM users in Hong Kong.

### Limitations

There are several limitations. First, the data collection was conducted online and promoted through social media, and so younger and educated individuals might be overrepresented in this study. This might lead to selection bias and neglect the participation of older individuals who had limited Internet access. Hence, paper-based surveys in the community and structured interviews would be performed to draw a concrete conclusion about the predictors of the intention to use TCM among Hong Kong population. Second, the recruitment criteria of participants, who had used TCM before, might lead to selection bias. Both TCM users and nonusers would be recruited in further research studies. Third, reliance on the self-report of the questionnaires might cause a measurement bias. Future research using qualitative study to investigate the predictors of the intention of using TCM is recommended. Finally, perceived needs to use TCM is also important in determining the intention to use TCM, but this topic was not included in the questionnaire. Further research should include the construct measuring individual perceived needs to use TCM.

## Conclusions

TCM has a long history in Hong Kong and is used by many people in the community. To our knowledge, this is the first study to investigate the predictors of the intention to use TCM in Hong Kong by using TPB. Individuals’ attitude towards TCM has been shown to have stronger effect on the intention to use TCM than other predictors, such as satisfaction, perceived behavioral control and subjective norms. Moreover, the indirect effect of the relationship of subjective norms, perceived behavioral control, knowledge, perceived service quality and intention to use TCM are supported in this study. To enhance the intention to use TCM in Hong Kong, key stakeholders should develop a positive attitude towards TCM in the population. In addition, it is suggested that researchers should explore the in-depth views on the intention to use TCM in future studies.

## Supplementary Information

**Additional file 1.** Supplementary file 1.

## Data Availability

The datasets used and analysed during the current study are available from the corresponding author on reasonable request.

## Abbreviations

ATT: Attitude
AVE: Average variance extracted
BI: Behavioral intention
HTMT: Heterotrait-monotrait
K: Knowledge
PBC: Perceived behavioral control
PSQ: Perceived service quality
SAT: Satisfaction
SN: Subjective norms
TCM: Traditional Chinese medicine
