# Supplementary material for: Predictors of the intention to use traditional Chinese medicine (TCM) using extended theory of planned behavior: a cross-sectional study among TCM users in Hong Kong
Source: BMC Complement Med Ther. 2022 Apr 22;22:113. doi: 10.1186/s12906-022-03598-x (PMC9028891; doi:10.1186/s12906-022-03598-x)
Supplement: Supplementary file 1 — Additional file 1. Supplementary file 1. [file 12906_2022_3598_MOESM1_ESM.docx]

**Questionnaire about your intention to use traditional Chinese medicine**

**使用中醫的意向問卷調查**

**Introduction to Survey**

**調查簡介**

The Centre for Ageing and Healthcare Management Research (CAMHR) at School of Professional Education and Executive Development (SPEED) of The Hong Kong Polytechnic University aims to investigate the intention to use traditional Chinese medicine in Hong Kong. Your response will help us to identify the predictors of the intention to use of TCM in Hong Kong. The questionnaire will take around 5 – 8 minutes to finish. To guarantee the accuracy of data, please avoid multiple entries by the same individual.

After the questionnaire is completed, your answers will be transmitted to the data collection platform embedded in the survey system, and the research team will conceal your identifiers. Information collected in this survey is strictly confidential and will only be used to broadly study for this study. Your personal identity would not be disclosed.

If you have difficulty in completing the survey, please email us at [spcahmr@speed-polyu.edu.hk](mailto:spcahmr@speed-polyu.edu.hk).

香港理工大學專業進修學院（PolyU SPEED）老齡化和醫療管理研究中心旨在研究香港大眾使用中醫的意向。你們的回覆將有助我們了解有甚麼因素影響香港大眾使用中醫。問卷約需5 - 8 分鐘完成。為確保數據的準確性，請你避免多次填寫此問卷。

問卷完成後，你的回答會傳送到調查系統內嵌的數據採集平台，研究團隊會隱藏您的身份信息。是次調查所收集的資料將嚴加保密，僅用於此研究項目。你的個人資料將不會被外洩。

如果你在完成問卷時遇到困難，請向我們發送電子郵件 ([spcahmr@speed-polyu.edu.hk](mailto:spcahmr@speed-polyu.edu.hk)) 。

Please check ✓ in the box for your approved consent of survey participation.

如您已明白及將會參與是次問卷調查，請在相應之方格填上“✓”號，以示同意。

🞏 I have acknowledged my right and agreed to participate in this survey voluntarily.

🞏 我已清楚我的權利並同意自願參加這項調查。

Are you a Hong Kong resident?

你是香港居民嗎？

🞏 Yes 是 🞏 No否

Have you used traditional Chinese Medicine (TCM) before? (TCM include Chinese herbal medicine, acupuncture, bone-setting, decoctions, gua sha, massage, moxibustion)

你以前用過中醫嗎？(中醫包括中藥、針灸、正骨、湯劑、刮痧、推拿、艾灸)

🞏 Yes 有 🞏 No沒有

1. The following questions are related to your attitudes on TCM, please check"✓" in the corresponding box.

1. 以下是一些有關於你對於中醫態度的題目，請在相應之方格填上“✓”號。

|  | Strongly disagree | Disagree | Somewhat disagree | Neutral | Somewhat agree | Agree | Strongly agree |
| --- | --- | --- | --- | --- | --- | --- | --- |
|  | 非常  不同意 | 不同意 | 有點  不同意 | 中立 | 有點同意 | 同意 | 非常  同意 |
| a. I extremely trust TCM.  a. 我非常相信中醫。 | 🞏 | 🞏 | 🞏 | 🞏 | 🞏 | 🞏 | 🞏 |
| b. I am very concerned about TCM.  b. 我非常關注中醫。 | 🞏 | 🞏 | 🞏 | 🞏 | 🞏 | 🞏 | 🞏 |
| c. I’d very much like to accept TCM theory.  c. 我非常願意接受中醫理論。 | 🞏 | 🞏 | 🞏 | 🞏 | 🞏 | 🞏 | 🞏 |
| d. I think TCM is effective.  d. 我認為中醫是有效的。 | 🞏 | 🞏 | 🞏 | 🞏 | 🞏 | 🞏 | 🞏 |
| e. I think TCM is safe.  e. 我認為中醫是安全的。 | 🞏 | 🞏 | 🞏 | 🞏 | 🞏 | 🞏 | 🞏 |
| f. I think TCM has few side effects.  f. 我認為中醫幾乎沒有副作用。 | 🞏 | 🞏 | 🞏 | 🞏 | 🞏 | 🞏 | 🞏 |

2. The following questions are related to your subjective norms on TCM, please check"✓" in the corresponding box.

2. 以下是一些有關於你對於中醫主觀規範的題目，請在相應之方格填上“✓”號。

|  | Strongly disagree | Disagree | Somewhat disagree | Neutral | Somewhat agree | Agree | Strongly agree |
| --- | --- | --- | --- | --- | --- | --- | --- |
|  | 非常  不同意 | 不同意 | 有點  不同意 | 中立 | 有點同意 | 同意 | 非常  同意 |
| a. My family and friends support me to choose TCM.  a. 我的家人和朋友支持我選擇中醫。 | 🞏 | 🞏 | 🞏 | 🞏 | 🞏 | 🞏 | 🞏 |
| b. My family and friends think I should choose TCM.  b. 我的家人和朋友認為我應該選擇中醫。 | 🞏 | 🞏 | 🞏 | 🞏 | 🞏 | 🞏 | 🞏 |
| c. My family and friends will choose TCM.  c. 我的家人和朋友會選擇中醫。 | 🞏 | 🞏 | 🞏 | 🞏 | 🞏 | 🞏 | 🞏 |
| d. My family and friends choose TCM, I would make the same choice.  d. 我的家人和朋友選擇中醫，我也會做出同樣的選擇。 | 🞏 | 🞏 | 🞏 | 🞏 | 🞏 | 🞏 | 🞏 |

3. The following questions are related to your perceived behavioral control on TCM, please check"✓" in the corresponding box.

3. 以下是一些有關於你對於中醫知覺行為控制的題目，請在相應之方格填上“✓”號。

|  | Strongly disagree | Disagree | Somewhat disagree | Neutral | Somewhat agree | Agree | Strongly agree |
| --- | --- | --- | --- | --- | --- | --- | --- |
|  | 非常  不同意 | 不同意 | 有點  不同意 | 中立 | 有點同意 | 同意 | 非常  同意 |
| a. I have time to receive TCM services.  a. 我有時間接受中醫服務。 | 🞏 | 🞏 | 🞏 | 🞏 | 🞏 | 🞏 | 🞏 |
| b. I am economically capable to receive TCM services.  b. 我有經濟能力接受中醫服務。 | 🞏 | 🞏 | 🞏 | 🞏 | 🞏 | 🞏 | 🞏 |
| c. I have the ability to decide whether to choose TCM.  c. 我有能力決定是否選擇中醫。 | 🞏 | 🞏 | 🞏 | 🞏 | 🞏 | 🞏 | 🞏 |
| d. I can share my knowledge and experience of TCM with others.  d. 我可以與他人分享我的中醫知識和經驗。 | 🞏 | 🞏 | 🞏 | 🞏 | 🞏 | 🞏 | 🞏 |
| e. I can overcome my difficulty in choosing TCM.  e. 我能克服選擇中醫的困難。 | 🞏 | 🞏 | 🞏 | 🞏 | 🞏 | 🞏 | 🞏 |

4. The following questions are related to your behavioral intention on TCM, please check"✓" in the corresponding box.

4. 以下是一些有關於你對於中醫行為意向的題目，請在相應之方格填上“✓”號。

|  | Strongly disagree | Disagree | Somewhat disagree | Neutral | Somewhat agree | Agree | Strongly agree |
| --- | --- | --- | --- | --- | --- | --- | --- |
|  | 非常  不同意 | 不同意 | 有點  不同意 | 中立 | 有點同意 | 同意 | 非常  同意 |
| a. I would like to use TCM in the future.  a. 我打算未來會使用中醫。 | 🞏 | 🞏 | 🞏 | 🞏 | 🞏 | 🞏 | 🞏 |
| b. I would recommend relatives, friends and colleagues to choose TCM.  b. 我會推薦中醫給親朋戚友和同事。 | 🞏 | 🞏 | 🞏 | 🞏 | 🞏 | 🞏 | 🞏 |
| c. I would say positive things about TCM to others.  c. 我會向別人說有關中醫正面的事情。 | 🞏 | 🞏 | 🞏 | 🞏 | 🞏 | 🞏 | 🞏 |
| d. I would encourage others to use TCM.  d. 我會鼓勵別人使用中醫。 | 🞏 | 🞏 | 🞏 | 🞏 | 🞏 | 🞏 | 🞏 |

5. The following questions are related to your perceived service quality of TCM, please check"✓" in the corresponding box.

5. 以下是一些有關於你對於中醫服務品質的題目，請在相應之方格填上“✓”號。

|  | Never | Seldom | Occasionally | Sometimes | Often | Usually | Always |
| --- | --- | --- | --- | --- | --- | --- | --- |
|  | 從不 | 不常 | 偶爾 | 有時候 | 常常 | 通常 | 總是 |
| a. The TCM services have up-to-date equipment.  a. 我使用的中醫服務擁有最新的設備。 | 🞏 | 🞏 | 🞏 | 🞏 | 🞏 | 🞏 | 🞏 |
| b. The TCM services provide their service at the time they promise to do so.  b. 我使用的中醫服務在他們承諾的時間內提供服務。 | 🞏 | 🞏 | 🞏 | 🞏 | 🞏 | 🞏 | 🞏 |
| c. Personnel of the TCM services react promptly to my requests.  c. 中醫服務的員工對我的要求有迅速作出反應。 | 🞏 | 🞏 | 🞏 | 🞏 | 🞏 | 🞏 | 🞏 |
| d. Personnel of the TCM services are polite.  d. 中醫服務的員工有禮。 | 🞏 | 🞏 | 🞏 | 🞏 | 🞏 | 🞏 | 🞏 |
| e. Personnel of the TCM services give me personal attention.  e. 中醫服務的員工有給予我關注。 | 🞏 | 🞏 | 🞏 | 🞏 | 🞏 | 🞏 | 🞏 |
| f. Personnel of the TCM services communicate carefully with me  f. 中醫服務的員工有認真地與我溝通。 | 🞏 | 🞏 | 🞏 | 🞏 | 🞏 | 🞏 | 🞏 |

6. The following questions are related to your satisfaction on TCM, please check"✓" in the corresponding box.

6. 以下是一些有關於你對於中醫滿意度的題目，請在相應之方格填上“✓”號。

|  | Strongly disagree | Disagree | Somewhat disagree | Neutral | Somewhat agree | Agree | Strongly agree |
| --- | --- | --- | --- | --- | --- | --- | --- |
|  | 非常  不同意 | 不同意 | 有點  不同意 | 中立 | 有點同意 | 同意 | 非常  同意 |
| a. My chief complaints can be addressed after receiving TCM.  a. 接受中醫治療後，我的主要症狀可以解決。 | 🞏 | 🞏 | 🞏 | 🞏 | 🞏 | 🞏 | 🞏 |
| b. I feel better after receiving TCM.  b. 我接受中醫治療後，我的感覺好了。 | 🞏 | 🞏 | 🞏 | 🞏 | 🞏 | 🞏 | 🞏 |
| c. TCM can fulfill my expectation of treatment.  c. 中醫可以滿足我對治療的期望。 | 🞏 | 🞏 | 🞏 | 🞏 | 🞏 | 🞏 | 🞏 |
| d. I am satisfied with the last curative effect.  d. 我對最近的療效很滿意。 | 🞏 | 🞏 | 🞏 | 🞏 | 🞏 | 🞏 | 🞏 |

7. The following questions are related to your knowledge on TCM, please check"✓" in the corresponding box.

7. 以下是一些有關於你對於中醫知識的題目，請在相應之方格填上“✓”號。

|  | Strongly disagree | Disagree | Somewhat disagree | Neutral | Somewhat agree | Agree | Strongly agree |
| --- | --- | --- | --- | --- | --- | --- | --- |
|  | 非常  不同意 | 不同意 | 有點  不同意 | 中立 | 有點同意 | 同意 | 非常  同意 |
| a. I know the basic theory of TCM.  a. 我了解中醫的基礎理論。 | 🞏 | 🞏 | 🞏 | 🞏 | 🞏 | 🞏 | 🞏 |
| b. I know the medicine (中藥) of TCM.  b. 我了解中藥。 | 🞏 | 🞏 | 🞏 | 🞏 | 🞏 | 🞏 | 🞏 |
| c. I know the diagnosis of TCM.  c. 我了解中醫的辨證。 | 🞏 | 🞏 | 🞏 | 🞏 | 🞏 | 🞏 | 🞏 |
| d. I know the diet of TCM.  d. 我了解中醫的食療。 | 🞏 | 🞏 | 🞏 | 🞏 | 🞏 | 🞏 | 🞏 |

8. What is your age?

8. 你的年齡是甚麼？

| 🞏 18 – 25歲 | 🞏 26 – 35歲 | 🞏 36 – 45歲 | 🞏 46 – 55歲 |
| --- | --- | --- | --- |
| 🞏 56 – 65歲 | 🞏 66 – 75歲 | 🞏 76 – 85歲 | 🞏 86 or above  🞏 86 歲或以上 |

9. What is your gender?

9. 你的性別是甚麼？

| 🞏 Male 男 | 🞏 Female 女 |
| --- | --- |

10. What is your highest education attained?

10. 你獲得的最高學歷是甚麼？

| 🞏 Primary or below  🞏 小學及或以下 | 🞏 Secondary  🞏 中學 | 🞏 Post-secondary  🞏 專上 |
| --- | --- | --- |
| 🞏 Degree  🞏 學士學位 | 🞏 Postgraduate or above  🞏 碩士或以上 | 🞏 N/A  🞏 不適用 |

11. What is your religion?

11. 你的宗教是甚麼？

| 🞏 Christianity  🞏 基督教 | 🞏 Catholic  🞏 天主教 | 🞏 Buddhism  🞏 佛教 | 🞏 Taoism  🞏 道教 |
| --- | --- | --- | --- |
| 🞏 Confucianism  🞏 儒學 | 🞏 Muslim  🞏 穆斯林 | 🞏 No  🞏 沒有宗教信仰 | 🞏 Others  🞏 其他 |

12. What is your current employment status?

12. 你現時的就業狀況是?

| 🞏 Student 學生 | 🞏 Employed 受僱 | 🞏 Self-employed 自僱 |
| --- | --- | --- |
| 🞏 Unemployed 無業 | 🞏 Retired 退休 | 🞏 |

13. Which categories describe your monthly income (in Hong Kong dollar)?

13. 你每月的收入(港元)是？

| 🞏 ≤ $4,000 | 🞏 $4,001 - $8,000 | 🞏 $8,001 - $12,000 | 🞏 $12,001 - $16,000 |
| --- | --- | --- | --- |
| 🞏 $16,001 - $20,000 | 🞏 $20,001 - $24,000 | 🞏 $24,001 - $28,000 | 🞏 > $28,000 |

14. Would you say your health in general is excellent, very good, good, fair, or poor?

14. 你認為你身體狀況是優秀、非常好、良好、一般還是差？

| 🞏 Poor 差 | 🞏 Fair 一般 | 🞏 Good良好 | 🞏 Very good非常好 | 🞏 Excellent 優秀 |
| --- | --- | --- | --- | --- |

15. Your prefix and first three digits of your HKID is (If your HKID is A123456(7), please fill in A123)

15. 你身份證上的字母及首三個數字是 (如你的身份證號碼是A123456(7), 請填上A123)

16. Email: *(optional for receiving results of this study)*

16. 電郵地址:  *(如希望收取本研究之結果，可選擇填寫)*
